# Adolescents and parents’ perceptions of best time for sex and sexual communications from two communities in the Eastern and Volta Regions of Ghana: implications for HIV and AIDS education

**DOI:** 10.1186/1472-698X-13-40

**Published:** 2013-09-26

**Authors:** Emmanuel Asampong, Joseph Osafo, Jeffrey Bart Bingenheimer, Clement Ahiadeke

**Affiliations:** 1 Department of Social and Behavioural Sciences, University of Ghana School of Public Health, Legon, Ghana; 2 Department of Psychology, University of Ghana, Legon, Ghana; 3 School of Public Health and Health Services, The George Washington University, Washington, USA; 4 Institute of Statistical, Social and Economic Research (ISSER), University of Ghana, Legon, Ghana

**Keywords:** Adolescents, Parents, Sex, Communication, Ghana

## Abstract

**Background:**

Adolescents and parents’ differ in their perceptions regarding engaging in sexual activity and protecting themselves from pregnancy and sexually transmitted infections (STIs). The views of adolescents and parents from two south-eastern communities in Ghana regarding best time for sex and sexual communications were examined.

**Methods:**

Focus Group interviews were conducted with parents and adolescents (both In-school and Out-of school) from two communities (Somanya and Adidome) in the Eastern and Volta regions of Ghana with epidemiological differentials in HIV infection.

**Results:**

Findings showed parents and adolescents agree that the best timing for sexual activity amongst adolescents is determined by socioeconomic viability. In practice however, there were tensions between adolescents and parents crystallized by spoilt generation and physiological drive ideologies. Whilst one community relied on a more communal approach in controlling their children; the other relied on a confrontational approach. Sex-talk is examined as a measure to reduce these tensions, and children in both communities were ambivalent over sexual communication between their parents and themselves. Parents from the two communities however differed in their perceptions. Whilst parents in one community attributed reduced teenage pregnancies to sex education, those in the other community indicated a generalized adolescents’ sexual activeness manifested in the perceived widespread delinquency in the community.

**Conclusion:**

Parents in both communities reported significant barriers to parents-adolescents sexual communication. Parents in both communities should be educated to discuss the broader issues on sexuality that affects adolescents and their reproductive health needs.

## Background

Adolescents’ participation in sexually risky behaviours is a source of concern to parents and adolescent health care providers worldwide [1]. In recent times, adolescents in Ghana are known to be sexually active and many believe their intimate friends are sexually active as well [2]. They engage in all sorts of sexual behaviours that can be attributed to the rapid social, cultural and economic changes occurring at both the local and international levels. It cannot also be denied that the HIV and AIDS epidemic has radically changed the world, yet many adolescents continue to engage in risky sexual behaviours [3]. For instance in a reproductive health study among secondary school students in the Upper East Region of Ghana, a disturbing low familiarity about family planning methods, as well as HIV and AIDS transmission was found among the students [4]. Besides, minimal contraceptive use puts them at high risk for unwanted pregnancies and sexual infections transmission.

The desire for people to have high levels of education has extended the period of adolescence [5]. In effect, one may be over and above the stipulated adolescent age range yet may be seen as an adolescent as long as he/she continues to live under the roof of his/her parents.

The age range of adolescents as defined by the World Health Organization is from 10 to 19 years [6]. This has been adopted by the Ministry of Health and the Ghana Health Service. The 2010 census conducted in Ghana indicated that adolescents formed 22.4% of the total population of 24,658,823 [7]. That makes it a significant portion of the populace that cannot be overlooked in relation to risky sexual behaviours that may put them at risk of contracting HIV and AIDS. All adolescents are definitely not alike. That is to say that they differ in demographic and social background. For example, their gender, age, level of education, socio-economic status, geographical location, ethnicity, sexuality, family circumstances just to mention a few may all differ from one adolescent to the other [8]. In a study of sexual behaviour among four sub-Saharan African countries including Ghana, findings indicated that adolescents have high levels of awareness but little in-depth knowledge about pregnancy and HIV prevention [2].

Adolescents’ decisions regarding engaging in sexual activity and protecting themselves from pregnancy and sexually transmitted infections (STIs) are influenced by many factors. For instance, in a study of students aged 13 to 18, it was reported that non- initiation of sex was associated with having a two-parent family and higher socioeconomic status, residing in a rural area, performing better in school, feeling greater religiosity, not having suicidal thoughts, and believing parents care and hold high expectations for their children [9]. In another study, adolescents who reported being highly satisfied with their relationship with parents were 2.7 times less likely to engage in sex than teens who had little satisfaction with their parental relationships [10]. In that same study, relationship satisfaction with parents was associated with a lower probability of engaging in sex, higher probability of using birth control if sex occurred, and lower probability of pregnancy during the ensuing 12 months. On the other hand, adolescents’ perception of maternal opposition toward engaging in sex was associated with a lower probability of engaging in sex and a lower probability of pregnancy during the ensuing 12 months.

Indeed the notion that parents have significant influence on the sexual and reproductive health of their children cannot be overemphasized [11]. It has been speculated for example that adolescents who are close to their parents may engage in less sexual activity because parent–child closeness increases opportunities for prosocial development [12]. There is also evidence to suggest that parent–adolescent communication about sex plays an important role in predicting adolescent sexual behaviour [13,14]. Besides, parental supervision and monitoring is said to be an effective way of controlling adolescents’ sexual behaviours. It is however important to note that the period of adolescence is characterized by shifts in influence, where peers become more influential than parents [15]. This situation is worsened by the tendency for parents to allow their adolescent children the freedom to spend increased unsupervised time with peers [16]. Furthermore, adolescents seek to acquire more insights into life skill-based sex education which is usually absent unlike parents who become more interested in moral education for their adolescent children [17].

In Ghana, anecdotal information indicates that talking about sex and sexual activities is considered a taboo for which reason parents have difficulties discussing anything related to sex with their adolescent children. Adolescents on the other hand cannot freely discuss sexual issues with their parents. This obviously is an affront to their right to free speech as guaranteed by the 1992 Constitution of the Republic of Ghana. In all these dynamics, parents’ perception about the best time for adolescents to engage in sexual activities is contrary to what adolescents perceive. These different perceptual positions manifest in certain entrenched opinions that are held by parents about adolescents and vice versa. Against the backdrop that specific cultural settings and different pathways in communities shape the sexual behaviors of young men and women [18], this paper seeks to shed light on the perceptions of best time for sex and sexual communications between adolescents and parents from two communities in Ghana. A further interest is to reflect on how these perceptions impact on sexual communication between adolescents and parents and the implications for HIV and AIDS education among adolescents in the country.

## Methods

This paper relied on a qualitative data initially generated from a longitudinal cohort study (LCS) of adolescents and their parents residing in two communities (Adidome and Somanya) in south-eastern Ghana. These communities are characterized by dramatic variations in localized HIV prevalence, allowing us to assess how epidemiological circumstances impinge upon adolescent sexual behaviour. In using the qualitative approach, the study specifically employed focus group discussions (FGDs) to investigate the perceptions of parents on the best time for sex for their adolescent children and examine the nature of sexual communication between parents and adolescents. Some of the items on the focus group discussion guide included the following: Who should educate the teenage boy or girl on issues relating to sex? Whose duty is it in particular to check the behaviour or conduct of adolescents between 13 and 18 years in this town? What should parents do if they realize that their under-age children are engage in sex? In reality, are parents talking to their children about sex, and if they do, how do they do it in this town? The FGD was conducted, one with mothers and female caregivers of adolescents, and one with fathers and male caregivers of adolescents in each of these two communities. FGD was also conducted with both In-school (males and females) and Out-of school (males and females) groups of adolescents in each of the two communities. Each FGD lasted for 90 minutes and a total of 12 interviews were conducted; one for each category of informants.

### Study settings

#### Somanya

The residents of the Manya Krobo and Yilo Krobo Districts of Eastern Region have experienced a particularly severe HIV epidemic. This is due in part to the circular migration of young women from this area to Abidjan, Côte d’Iviore, where many engage in commercial sex work—a phenomenon that dates to the displacement of populations by the creation of Lake Volta during the 1960s [19,20]. The severity of this localized epidemic is reflected in sentinel surveillance data. The antenatal clinic in Somanya of the Manya Krobo District, has consistently recorded the highest levels of HIV prevalence among all 40 of Ghana’s sentinel surveillance sites; in 2006, prevalence at Somanya was 8.4%, compared to the next highest prevalence of 5.6% and the national average of 3.2% [21]. Epidemiological studies in the area also demonstrate very high levels of HIV prevalence. At the Somanya hospital, 23% of male and 17% of female outpatients seen in the year 2000 were HIV-positive, while another report indicated an HIV prevalence level of 18.5% among women attending antenatal clinics in Somanya and nearby Atua in 1999 [22,23]. Somanya is a regional trading centre about 30 km south of the Akosombo Dam. The main road from Accra to Akosombo passes through it, and smaller roads branch out in several directions to rural villages and farmland.

#### Adidome

It is located in the North Tongu District, just 25 km to the northeast, the town of Juapong, in the North Tongu District of Volta Region, and has much in common with Somanya. It is a regional trading centre located on a major road, surrounded by rural villages and farmland along smaller roads in several directions. In contrast to Somanya, however, this area remains largely untouched by HIV. Among the 805 women attending the antenatal clinic at the North Tongu sentinel surveillance site in 2005 and 2006, none tested positive for HIV [21]. No other site in Ghana recorded a prevalence estimate this low during that period.

### Access to communities

Authorization was sought from local authorities mainly chiefs, elders and elected officials. The purpose of the study was first explained to officials in the two communities. Subsequently, the study began with a community event (a durbar where community members are assembled by a crier for some dissemination of information) in both localities. During these durbars, traditional and modern authorities (e.g., chiefs, elders, district assemblymen, and clergy members) announced the purpose of the study. In line with ethical principles, community members were not coerced and induced into participating in the study. They were told that they could opt out in the process if they found the study uncomfortable. During each phase of the study, community-wide events were held where community members received refreshments (e.g., food and drink). This was to provide the opportunity to interact with members of the research team as well as provide positive publicity for the study and serve as a method of community-level compensation.

### Ethical issues

Ethical approval for the study was sought from the Institutional Review Board (IRB) of the Noguchi Memorial Institute for Medical Research. Reference: NMIMR-IRB CPN 006/09-10. For all adult participants who agreed to be part of the study, a written informed consent was obtained from them. However, adolescents (who were out of school) and below 18 years sought clearance from their parents/guardians whilst those in school were cleared by the school authorities.

### Participants

In order to ensure gender equity, both female and male participants from the two communities took part in the study. In effect, participants in the study included mothers of adolescent children, female caregivers of adolescents, fathers of adolescents and male caregivers of adolescents. Adolescent participants were made up of In-school boys (ISB), In-school girls (ISG), Out-of school boys (OSB), and Out-of school girls (OSB). This was also to ensure that information received takes into account a gendered perspective. For purposes of convenience and anonymity, participants who showed interest in the study were asked to contact the researchers to be a part of the focus group that was made up of 6–8 members for each group.

### Analysis

Thematic analysis was used to generate themes. This method of analysis is used to identify, analyze and report patterns within data as well as interpreting various aspects of the research topic [24]. One of the benefits of thematic analysis is its flexibility, and can be applied *across* a range of theoretical and epistemological approaches [24]. The analysis started by looking for patterns of meaning and issues of potential interest in the data and organizing them into meaningful coding schemes or groups. This was followed by sorting the different codes into potential themes. A theme was not necessarily dependent on quantifiable measures – but in terms of whether it captures something important in relation to the overall research question. Themes were finally refined, defined, logical connections established between them and interpreted. Three themes were accordingly identified thus: *The Timing Defined, The Tension, and The Talk.* We did not observe differences in perceptions across age, sex and socioeconomic status of participants. For purposes of simplicity the following abbreviations shall be used to refer to participants as follows: out-of school boys (OSB), in-school boys (ISB), in-school girls (ISG), out-of school girls (OSG).

## Results

### The timing defined

This theme examines the perceived age at which sexual activity could start. Generally, parents from both communities pegged the age between ages 18-35 comparatively similar to those of adolescents 18-30. Again, parents from both communities seemed to offer a socioeconomic definition of maturity at which sexual behaviour is permitted. Voices of parents from both communities illustrate this below:

For me, 30 years is the best. At this age one is likely to be mentally and financially ready for marriage so that even if your very first sexual contact with a woman turns into a pregnancy, there will not be any problem (FGD, Father Adidome).

I think they should rather wait till they are 25 yrs old. By then he would have been working so the girls can start at 18 yrs and the boys 25 yrs (FGD mother Somanya).

Adolescents shared similar understanding from both communities as illustrated: “*It’s when you are working and you have your own money that you can have sex*” (*FGD, ISB Somanya*,). Another adolescent from Adidome said “*I think 20 years because at that time if he impregnates the girl, he can work to cater for both the girl and the pregnancy (FGD, OSB Adidome).*

From the above quotes, the perceived best time for sex for both adolescents and parents is socioeconomically determined. A person was thought of as ready for sex on condition that he has demonstrated economic ability or viability to cater for the woman and her pregnancy.

An interesting aspect of the perception of best time for onset of sex was the sense of patriarchy that undergirded the socioeconomic view. As could be noted in the above quotes, parents did think of marriage as more of a man’s (or boys) affair than women (or girls), and throughout, the discussion tended to dwell more on defining best time for sex for boys more than girls. In other words, the definition of age at which adolescent girls were perceived ready for sex was not clearly explained by parents.

However, adolescents did provide clear factors influencing the best timing for sex for adolescent girls. One such factor was the quest for *economic independence*. We illustrate this independence in the conversation below:

Q: By what age should a girl first have sex?

R: I think 30…By then you are fully mature and will be having your own job (FGD ISB Adidome).

R: I think 23 years, by then you will have completed school and got yourself a job and thus can cater for pregnancy (FGD ISB Adidome).

By age 25, by then you‘d have had a job so that if the man abandons you can take care of yourself (FGD ISG, Adidome).

The above view is corroborated by another adolescent from Somanya thus:

“*They have to wait to have sex because if you have work that is if you are employed, when the boy misbehaves after pursuing you and dumping you, you can rely on your work to be able to look after yourself” (FGD, OSG Somanya).*

The economic independence as indicated by these adolescents might reflect a paradigm shift from the prevailing patriarchal view that women ought to depend on men for survival to a more contemporary view of women empowerment practices around the world.

### The tensions

Although adolescents and parents agreed in principle that the age of onset for sexual activities is socioeconomically constructed, in practice, they sharply disagree with each other as to when adolescents should first have sex. In both Somanya and Adidome, this view prevailed among adolescents:

They don’t agree because at this age your parent can’t tell you to go and have a boyfriend but at this age the boy will be feeling like having sex but the mother will not agree. If the mother asks the boy to reach 25 years before having sex, the boy will not agree (FGD ISB Somanya).

A parent from Somanya corroborates this

…*they do not agree with us because they even start before they reach that age. The child cannot tell you that they don’t agree but since they are starting early, it means they are not in agreement concerning that age” (FGD mother, Somanya*).

In Adidome, the story is not different. There seems to be tension between children and parents regarding age of onset for sex as indicated below:

“*Now, modern day boys and girls are in a hurry to have sex, if it were not so, a thirteen year old girl would not be pregnant. In the past, virginity was cherished and even verified when one got married. But now it is not*” (*FGD, father Adidome*).

An adolescent in Adidome corroborates this thus: “*Some don’t agree, they get angry and they say to their parents that their peers are doing it”* (*FGD ISG Adidome*).

This tension over timing of sex between parents and adolescents seems aggravated by two key perceptions. The first is the “*spoilt generation ideology*” and the “*physiological drive ideology”.*

#### The “spoilt” generational ideology

This was held by parents and they described adolescents as lacking morals for proper behaviour. This was done by comparing their (older) generation which they claimed was often responsive to parental orders and subservient to social prohibitions:

In our time, there was respect for mothers and children listened to their parents but now, they do not respect nor listen to their parents anymore so there is a big difference. (FGD mothers Somanya).

Parents in Somanya seemed to hold a negative attitude about their adolescent children describing them in very negative terms undergirded with some anger. They used terms such as ‘spoilt’, ‘bad’, ‘people with no morals’, ‘dangerous’ and ‘shameless’ to describe adolescents:

They are shameless (chorus), yes we see them as shameless people (interjection: we see them as bad children). They are irresponsible and shameless, doing things anyhow. If you say it, you will be insulted (FGD, father Somanya).

In Adidome, parents equally described their children as disrespectful but did not appear to denigrate them as we saw in Somanya. A parent in Adidome said:

But nowadays, the children are bad and disrespectful. Some of the children even send their partners to their parents’ bed when their parents have travelled, a girl will take a boy to the parents’ bed. In the past, you can’t do that because your parent will sell the bed to you when you are caught. (FGD mother, Adidome).

In response to whether adolescents were engaging in early sex in Adidome, a parent said: *“They may be doing it, but then, they do it with some respect” (FGD, father Adidome).* Engaging in sex respectfully is not explained but it might essentially illustrate the non-derogatory view parents held about adolescents in the Adidome community.

In general adolescents seemed aware of this ‘spoilt’ generational ideology of parents about them: *Our parents think that having sex at this age is not good because when you have sex you do not respect anybody (FGD ISB Somanya).* This is also corroborated by another adolescent in Adidome: “*They say the child is disrespectful, that with this promiscuous life she’ll get pregnant too early. Others say they’ll contract STDs (FGD, ISG Adidome).*

#### The “physiological drive ideology”

This view was emphasized by adolescents from the two communities. By this adolescents expressed the view that their decision to engage in sex is also often driven by pure hormonal or biochemical influences in their bodies:

It is not easy to wait because by the age of 15 years going, our organs disturb a lot. Especially in the morning, you will feel ejaculations so through that you will be thinking of having sex and this can lead you into having early sex (FGD, ISB Somanya).

This view is corroborated by adolescents in Adidome:

I agree with the one who said adolescent age, which is 13 years. This is because when you get to that stage you will be experiencing some feelings, which will be pushing you into that. Therefore, you have the feeling in your body that you are old enough to have sex. (FGD, OSB Adidome).

The tensions between parents and adolescent seem crystalized within these ideologies. Parents think adolescents are unlike those of yesterday and do not understand the rules and orders that have regulated and built moral behaviours of children in the past and perhaps reduced sexual infections. Adolescents on the other hand seemed to perceive parents as perhaps ignorant of the biochemical influences in adolescence which makes their experience unique and thus justifiable if they want to have early sex than expected.

These tensions seemed to have generated the use of control measures on the part of parents to regulate adolescents’ early desire to have sex. Adolescents were either aware of such measures and did confirm them or report other measures which were not reported by parents. For instance whilst adolescents in Somanya reported (n = 7) their parents relied more on ***partner-parental caution*** (this is where parents did warn the parents of their wards’ partners to restrain their children) as in: “*One of the parents will go and confront the girl’s parents to warn the girl to stop seeing their son” (FGD, Somanya, ISB*); parents in Somanya did not seem to clearly show what they do. They appeared to recommend what *should be done* than what they *are doing.* Thus they indicated that parents *should rely* on ***parental counsel*** to regulate adolescents’ early sexual behaviour. For instance:

“*You have to call the child gently and talk to her…… Mothers should be most responsible for their kids’ life. We should be closer to the children*” (*FGD, mother Somanya*).

The partner-parental caution could foster interpersonal relationship difficulties between parents and thus might not auger well for any concerted effort to control adolescents’ sexual behaviour in Somanya.

The situation seemed different in Adidome. Here children reported that parents relied on ***institutional collaboration*** (where they appealed to other social institutions such as the police, teachers/schools, churches/religious leaders) to help regulate the sexual behaviour of adolescents:

*“Some parent may report to teachers. Some parents report to the police station and say their daughter is in their hands/the hands of the boy’s family with the police witnessing. They say whatever happens that family is responsible”* (FGD ISG Adidome).

Parents from Adidome confirmed relying on a strong presence of ***communal socialization*** undergirded by proscriptive morality. In such sociocultural space, raising children is not viewed as a sole responsibility of parents to train their children but rather a communal one:

*“Now we have some committee in this town and they watch over the town in the night so if they get hold of a young man or young woman in the night, they beat them so they will stop doing bad things in town”* (FGD mother Adidome).

A father in Adidome endorses this: “*In these areas, every parent disciplines a child when they go wrong. There is nothing like he is not my son or daughter so I cannot punish her” (FGD, father, Adidome).*

### The talk: sexual communication

In this theme we analyze whether sexual communication occurs between adolescents and parents as perhaps a potential measure to reduce the tensions between adolescents and examine barriers towards sexual communication.

The general view was that adolescents from the two communities were ambivalent as to whether sex talks did occur between them and their parents. Whilst some reported that parents were not doing that: “*They don’t talk to us; they say when they talk to us about it sex we will go and practice it, and that when they talk to us about sex it, they will be spoiling us and therefore they don’t tell us anything’. (FGD ISG, Somanya).* Others thought parents were doing that “*There are others who discuss sex with their parents and their parents advise them on that” (FGD OSB, Somanya).*

The situation did not seem different in Adidome either. Some parents did engage their children in sex talks as reported by adolescents:

Some parent will study the way you behave and dress and when they realize you are into girlfriend/boyfriend relationship, they will then advice you to put a stop to it, but if your parents observe you critically and realize that you are of good character, I don’t think they will call to advice you (FGD ISB, Adidome).

However, other adolescents reported that sexual communication was not a common place in Adidome:

It's not common here. I haven't seen it being done. Sometimes the child may think if he/she goes to the parents, she'll be told that she's spoilt, or when she's talking then the parents will get annoyed with him and this makes the child afraid or scared to approach the parents. (FGD OSG Adidome).

Parents from these two communities do accept that sex talk was their responsibility but differed in opinions about whether parents did talk to their children about sex. In Adidome for instance fathers reported that the low rate of pregnancy relative to other neighbouring towns was an evidence of effective sexual communication between them and their children:

I will say they are doing it. The fact that there are not many instances of teenage pregnancies attests to the fact that they do it. They do it by not allowing their wards to go out at night and also checking and deciding the type of friend their children keep. Even if the children are engaged in sex, so far as there not many pregnancies seen, it is presumed that the parents are educating them on sex issues (FGD Father, Adidome).

A mother in Adidome also seems to emphasize this view of perceived effective sex talk as perhaps influencing children’s academic progress:

I have male children and they like to engage in relationship with girls so at times, I call them so we can have a conversation then I start talking about relationship then they will start laughing and I tell them that this is not something they should laugh about and that if at their age they get involved with girls, It will not be in their interest and now they are all now in secondary school (FGD mother, Adidome).

However, in Somanya it appeared that parents were not doing sex talk with adolescents. For instance fathers in Somanya unanimously (n = 7) indicated that sex education is not common in Somanya:

I don’t think that the education is on-going because while the father is telling the children to stop what they are doing, the mother will also call the children and say to them don’t mind your father (FGD, father Somanya).

It is true because parents in the majority, about 95% do not do that…today I said the parents have also shirk their responsibility in many ways (FGD, father Somanya).

Mothers in Somanya also could not clearly report that they do sex talk with their children. They rather do accept that it was the responsibility of parents and showed how it could *be* done:

“*Yes it is our responsibility. You have to for example teach them how to take care of themselves when they are menstruating and others. Tell them they will get pregnant when a man touches them. So most of us parents have to talk to our children about sex so they know the dangers associated with it*” (FGD, mother Somanya).

There was rather a pattern of perceived widespread adolescent delinquency and lack of communal socialization that parents in Somanya reported which could discourage anyone to talk to adolescents about sex (n = 7 out of 8 members in the group):

It is the absolute truth. If you should see your sister’s child misbehaving and you report to the mother, the mother will tell the child that you came to report him and that will be the end of you. They will either shoot you or inflict knife wounds on you (stab you). So you can only talk about it or punish the person if it is your own child. (FGD, Somanya mother).

In addition, supposing you see somebody stealing from a house and report those people, your identity will be revealed to them and they will conspire and ambush you and kill you. Because of that when you see any bad thing going on, you cannot talk about it. (FGD, mothers Somanya).

Fathers in Somanya seemed to indicate that this perceived widespread delinquency among adolescents is epitomised in the sexual activeness of children:

On Monday mornings come and look around the environment. What is condom? Sometimes they are pined to the wall and you’ll be seeing them. And yet when you say it they say; ‘foolish man at this time won’t you go to bed?’ a child of 6 years or 7 years. So I would say they are 2000% sexually active. (FGD, father Somanya).

*Right now the children are giving birth early. In the past, like my brother said, even at 18 a lady was still a virgin. But there is a girl in our area, who is 9 years but has given birth; another one is also 13 years and has given birth* (FGD, father Somanya).

Certain barriers to adolescent-parents sex talk were identified by both parents and children. As indicated above by the adolescents, the lack of effective sexual communication between them and their parents are saddled with significant barriers. Such barriers reported included the following:

#### Poor role models

Here, adolescents and parents did report that parents themselves were culprits of the very same sexual impropriety they desire their children to desist from, or for purposes of economic gains parents do not discuss sex:

“*Some parents who are into such activities who even try to advise their children against, have the children say “you are doing it, why are you telling me not to do it*” (*FGD, ISG Adidome).*

“*Also, if the boy has money and buy gifts for the girl’s parent, they will allow the relationship to continue because they know that they will be getting money from the boy. Some parents are part of the problem” (FGD, OSB Somanya).*

Parents in both communities did affirm such practice as reported by adolescents:

“*Some women don’t have husbands but have boyfriends and they send their girl child to go and collect money from their boyfriends. By the time this child gets to about 15 or 16 years, she is already spoilt. This leads to the children becoming ‘bad’ early*” (FGD, mother Adidome).

“*I’ll come in again; my friend; there are bad adults too among them, even those old enough to be fathers to these children. They’ll come and you’ll see them buying drink for these children. And after that they won’t even take them home, but as I said earlier they pin them to the walls. You see them from 11 pm onwards in the dark corners ……… so adults are also among*” (FGD, father Somanya).

Implicitly, adults are supposed to be guides and models for children. As indicated above, adults in Somanya actively patronise sexually active adolescents which could reduce the moral courage they might require to do sex talk with adolescents.

#### Taboos

Adolescents in Somanya did indicate that taboos were barriers to sex-talk between them and their parents:

“Some *do, a few. Not everyone does that. The few are usually those who are older about 20 years and above. As for our age group, we can’t. We are unable to. It is because, what you are told not to do, if you dare talk about it, you are doomed*” (FGD, OSG Somanya).

Another adolescent said “*Sometimes she will drive you away that you are not yet of age to talk about such things (FGD, ISG Somanya).*

These statements indicate age differentials at which children can communicate with parents about sexuality. It seems that there is a social proscription against sexual behaviours for younger age group and such social censure discourages free communication on the subject with parents. Sexual taboos were not mentioned by adolescents in Adidome as a barrier towards sex talk with parents.

Parents in Somanya did not identify any taboo as a barrier to sex-talk, but rather lamented about the Dipo, a rite that seems to have lost its traditional appeal and role as perhaps an effective system regulating sexual behaviours:

Right from birth she is trained from home. That is the responsibility of a woman so that before they reach the Dipo stage, she already knows all she should do as a woman because she has been prepared for marriage which is a godly institution. But today we say we are pastors and we have spoilt everything and spoilt the children, because we say it’s fetish but it’s not. So when a child is growing then parents cover her up for 2-5 years then they say she has performed the rights. In those days if you don’t do the Dipo and you get pregnant, they will sack you. So there was fear and the children also ……………… their bodies therefore become matured for marriage and child birth. (FGD, fathers Somanya).

In Adidome, adolescents did not report taboos as barriers, but parents in Adidome did:

“*Here in Tongu, anything related to sex is considered bad language- vulgar. Any time a child wants to talk about this, he or she is shut down. So, it never is the duty of parents to talk to their children about it. So children are not guarded against its effect*” (FGD father, Adidome).

Another parent in Adidome also corroborates this view “*Talking to children about sex is a dreaded topic here in Adidome. But I think ideally, it is the duty of the parents to do so*” (FGD father, Adidome).

#### Fear

Adolescents reported some level of anxiety in their attempt to approach parents on sexual issues: “*Some do, others don’t. Those who don’t are afraid of their parents; they think when they ask about sex they’ll be thought of as bad by their parents*” (FGD, ISG Adidome). Another child from Somanya corroborates this as “*No! because I am afraid that if I ask them about sex they will be annoyed with me. They will think that I am practising it”* (FGD, ISB Somanya).

Here, the fear is about misrepresentation. Adolescents usually have a strong sense of positive self-image [25], and it seems that they guard against tarnishing this image.

#### Exposure to technology

This view was emphasized by parents that adolescents’ unhindered access to technology denies parents the chance to do sex talks:

“*What I have observed is that when you think this child is now of age so I can advise her concerning sex, by that time, he/she already knows about it in a movie and has seen all the act on TV and even on radio stations. So when you call them to advise them they don’t take it.” (FGD, mother Somanya).*

This view was corroborated by another parent in Adidome:

I have also realized that in the past, there was no TV but nowadays, they show all kinds of things on the TV and the child will be watching. This is making the children to get spoilt early (FGD mother, Adidome).

In the above statements, parents view the electronic media as channels through which adolescents learn about sexual activities thus making parents’ role of providing sex education redundant. The challenge for them is rooted in the early exposure to what they perceive as volatile information to children without caution. In that sense, parents viewed the child as armed with information that could expose parent’s ignorance when they attempt to educate them about sex.

## Discussion and conclusion

The purpose of this article was to examine parents and adolescents’ perceptions about sexual timing and sexual talks as possible psychosocial factors that could influence the reported epidemiological differentials in HIV prevalence between Somanya and Adidome. In principle, adolescents and parents’ view of best timing for sexual behaviour for adolescents is socio-culturally constructed.

This theoretical perception of timing does not translate into practical behaviours in adolescents. Adolescents thus disagreed with their parents about onset of sexual behaviour. This tension seems crystalized in the way parents perceived adolescents-as tainted generation and how adolescents also perceived parents as ignorant about the biochemistry in adolescence. In several communities in Ghana, many parents frown upon sexual activity of their children outside the context of marriage; especially so when these children live under the roof of their parents. The wish of parents is for their adolescent children to be biologically mature, complete their education, gain employment after which one can think about getting married which is an official license to engage in sexual activities. This is usually cherished and regarded as an honour to the parents, the children themselves and the entire family. Anything short of that is deemed disappointing and in some instances a disgrace to the family especially when pregnancy occurs.

To this end, the expectation is for parents to provide supportive environments in which their adolescent children can have access to useful information taking into account an understanding of the development of their sexuality so that they do not engage in an untimely risky sexual behaviours. Children are first nurtured at home when it comes to adolescent sexuality [26]. Parents thus become the primary and significant influences on their children and have tremendous impacts on their attitudes and behaviours [27,28].

The issue of hormonal changes impacting on the sexual drive of adolescents cannot be wished away because those are natural dynamics that occur in every adolescent’s development. This fact was emphasized by adolescents in the two communities. The control mechanisms put in place however go a long way to minimize the extent and the time to which these adolescents engage in sexual activities. For example, in Somanya, it was observed that parents resorted to their individual personal effort in preventing their adolescent children from engaging in sexual activities whilst in Adidome, parents in addition to their personal efforts also engaged the services of other people and institutions to help control their adolescents’ sexual behaviours. This is consistent with our earlier findings where parents in Adidome relied on communal socialization to regulate sexual behaviours of their children [29]. The expectation therefore is for adolescents in Adidome to be better controlled than their counterparts in Somanya.

Talking about sexuality in many African cultures is perceived as a taboo, allowing only ceremonial rites or authorised persons to discuss the subject with young people [30]. In many countries however, these traditional ways of communicating sexual matters between generations have broken down due to lifestyle changes [31]. Traditionally, female adolescents were educated by aunts on sexual behaviours when they go into marriage, but aunts are no longer playing that role [32]. Unfortunately, these personalities including many parents have unintentionally reneged on this responsibility.

This can in part be attributed to the sensitivity surrounding sexual talk that makes parents uncomfortable when they have to discuss sexuality with their children. In instances where parents have discussed sexuality issues with their children, these discussions have often been incomplete [33]. Messages that are transmitted by parents therefore tend to be ambiguous and vague. This perhaps may be in consonance with the sexual mores in Ghana where reference to sexuality-related issues are done using euphemisms. In many cases, children neither understand these terminologies nor are they able to ask their parents for detailed explanations or seek clarifications on such issues. This is to avoid a situation where parents may confirm their perception of the children as spoilt.

Nevertheless, communication is said to be a two way process but in many Ghanaian communities, communication on sexual issues is usually done by parents who directly talk to their adolescent children, resorting to didactic and authoritative approaches [34]. In this instance all the adolescent children do is to listen and not to ask questions or talk back. The notion is held that a child who talks back to his/her parents is disobedient and so for most children, hardly would they want to play their role of being active partners in an effective communication process. What then happens is a unidirectional type of communication where it is only the parents who give the information [35].

It is therefore not surprising that, many adolescents receive most of their information on sexuality from their peers, which often leads to misinformation. Adolescent children who tend to be involved with friends end up in social contexts that encourage early dating and entry into romantic relationships [36]. Consequently, sexual initiation takes place earlier than anticipated [37].

In conclusion, sexual communication between adolescents and their parents in these two communities was a challenge. However there were differentials in the way this challenge was dealt with by parents in these communities. Whilst those in Somanya were antagonistic and held a negative view of their adolescents’ sexual behaviours, those from Adidome were more open minded and involved all relevant stakeholders in handling this challenge. Adolescents’ view of best time for sex and its communication could thus be largely situated within the biological discourse compared to the socioeconomic paradigm of parents. This could fuel the seeming tension between parents and adolescents and thereby hamper smooth sexual communication.

### Implications for HIV education in Ghana

The findings of the present study have important implications for HIV education programmes in Ghana: First, messages from parents must be clear and concise, devoid of warnings about the negative outcomes of pre-marital sex. Parents should thus discuss the broader issues on sexuality, to include adolescents’ sexuality and their reproductive health needs. Failure to provide adolescents with accurate information on these specific topics may place the adolescents at risk for negative outcomes, particularly if they seek such information from peers. In addition, parents need to adopt an open and receptive approach when initiating conversations, encouraging questions from adolescents and responding to these questions. An open process of sexuality communication involves both parents having adequate knowledge, being willing to listen, talking openly and freely, and understanding the feelings behind any questions posed by adolescents. This is to avoid the unidirectional process where only parents do the talking.

It is also important that institutions such as the Planned Parenthood Association of Ghana (PPAG) that is known to be involved and conversant with adolescent reproductive health issues be encouraged to announce their presence in communities where they can be of immense help to adolescent who otherwise would have difficulties engaging in sex talk with their parents. By so doing, they would get the requisite information that will help them take control of their sexual life especially. Adolescents cannot be denied that right which was emphasized at the International Conference on Population and Development (Cairo, 1994 and New York, 1998) as well as the Fourth World Conference on Women (Beijing, 1995), both coordinated by the UN, that affirmed the sexual and reproductive rights of young people.

### Limitation of study

This study has some limitations. The first is that it is case-driven and thus the findings cannot be generalized to every community in Ghana. Another is that the use of focus group discussion could discourage some parents from sharing other sensitive issues about their wards. Against the backdrop that within the interdependent social milieu in Ghana, a member’s misconduct could affect others, some parents are likely to hold back other information that might be sensitive, although useful and richer for analysis.

## Competing interests

The authors declare that they have no competing interest including financial to report.

## Authors’ contributions

EA: Interpretation of data and drafting of script. JO: Interpretation of data and drafting of script. JBB: Conceived study, concept formulation, and drafting of script. CA: Drafting of script. All authors read and approved the final manuscript.

## Pre-publication history

The pre-publication history for this paper can be accessed here:

http://www.biomedcentral.com/1472-698X/13/40/prepub
